# Health-Related Quality of Life in pre-dialysis patients with chronic kidney disease: the role of Big-Five personality traits and illness denial

**DOI:** 10.1186/s40359-022-00992-5

**Published:** 2022-12-10

**Authors:** Daniele Pugi, Fabio Ferretti, Maddalena Galeazzi, Giacomo Gualtieri, Lore Lorenzi, Niccolò Pappalardo, Pasquale Giuseppe Macrì, Guido Garosi, Anna Coluccia, Andrea Pozza

**Affiliations:** 1 Department of Medical Science, Surgery and Neurosciences, University of Siena, Viale Mario Bracci 16, 53100 Siena, Italy; 2 Department of Cardio-Thoraco-Vascular Sciences and Public Health, School of Specialization of Legal Medicine, University Hospital of Padova, Padua, Italy; 3 Santa Maria Alle Scotte University Hospital of Siena, Siena, Italy; 4 Family Therapy Institute of Bologna ITFB, Bologna, Italy; 5 UOC Medicina Legale e Area Azienda USL Toscana Sud Est, Arezzo, Italy; 6 UOC Nefrologia, Dialisi e Trapianti, Santa Maria alle Scotte University Hospital, Siena, Italy; 7 Psychology Unit Santa Maria Alle Scotte University Hospital, Siena, Italy

**Keywords:** Renal disease, Quality of life, Personality, Anxiety, Illness denial, Chronic disease, Anxiety, Big-Five personality

## Abstract

**Background:**

Health-Related Quality of Life (HRQoL) in patients with chronic kidney disease (CKD) is significantly affected, regardless of the stage of the disease, as regards the physical, psychological and social functioning dimension. Big-Five personality traits can affect patients’ HRQoL and willingness to take treatment options. Illness denial consists of denial of negative emotions, resistance to change and conscious avoidance. Poorer HRQoL can predict a higher risk of hospitalization and mortality, and broadly a worse adjustment to the dialytic therapy. Thus, a clearer knowledge of the psychological variables associated with a worse HRQoL in the predialysis stage might improve the intervention planning. No study investigated illness denial and personality traits simultaneously. We investigated the role of illness denial and Big-Five personality traits in the domains of HRQoL in predialysis patients with CKD.

**Methods:**

One hundred adults (mean age: 75.87 years) with CKD participated. The Kidney Disease Quality of Life Short form, the Italian version of Ten Item Personality Inventory Revised, the Illness Denial Questionnaire, and the State-Trait Anxiety Inventory Form-Y were administered.

**Results:**

Illness denial was associated with increased HRQoL related to symptoms/problems, effect and burden of CKD and cognitive functions domains, and it was a predictor of higher HRQoL in the last three domains mentioned above. Extraversion was related to better work status and sexual function; agreeableness was linked to elevated cognitive function, quality of social interaction and sexual function; conscientiousness was related to better sexual function; neuroticism was linked to improved cognitive and sexual functions; in the end, openness to experience was related to fewer symptoms and problems.

**Conclusions:**

This is the first study which simultaneously assessed Big-Five personality traits and illness denial in different domains of HRQoL of CKD patients. Personalised psychological interventions aimed at improving HRQoL in this population might focus on specific illness denial processes and personality traits.

## Background

### Health-Related Quality of life in chronic kidney disease

Health-Related Quality of Life (HRQoL) refers to the dimensions of functioning that are affected by one’s disease and/or treatment, including physical (the ability to perform activities of daily living, as well as physical symptoms resulting from the disease or treatment), psychological (ranging from severe psychological distress to a positive sense of well-being and cognitive functioning), and social functioning (quantitative and qualitative aspects of social relationships and societal integration) [1].

Chronic diseases (e.g., cancer, heart diseases, stroke, diabetes, bowel diseases, renal disease, and psychiatric diseases) have the potential of affecting and worsening the overall health of patients by limiting their capacity to have a good functional status and reducing the positive reinforcing outcomes of participating in valued activities and feelings of personal control [2–7].

Chronic kidney disease (CKD) is defined by indicators of kidney damage—imaging or proteinuria (i.e., albumin to creatinine ratio)—and decreased renal function—below thresholds of glomerular filtration rate (GFR) estimated from serum creatinine concentration—for at least three months [8].

The current recommendations by the Kidney Outcomes Quality Initiative [9] and National Institute for Health Excellence [10] are to use serum creatinine concentration to estimate glomerular filtration rate and transform it using the Chronic Kidney Disease Epidemiology Collaboration equation.

CKD compromises the physical—complications include anaemia, reduced red blood cell survival, iron deficiency, and mineral bone disease—and psychological health of patients, daily functioning, general wellbeing, and social functioning [11].

With a prevalence in the general population around 13% [12] CKD is associated with HRQoL across all the stages [13–15]. Poorer HRQoL is also related to a higher risk of developing end-stage kidney disease, which in turn predicts hospitalization and mortality, and falls as GFR declines [16]. The more the kidney function worsens, the more the risk of death increases, and it’s largely attributable to death form cardiovascular disease and cancer [17].

### Big-Five personality traits in CKD

The Big-Five model of personality integrates most of the evidence in personality psychology [18, 19] and assumes that individual differences in personality characteristics can be organised into five broad trait domains: extraversion (extravert people experience high levels of happiness and life satisfaction), agreeableness (individuals high in this trait are helpful, warm and emphatic), conscientiousness (persons high in this trait tend to be well-organised, goal-directed and persistent), neuroticism (persons high in this trait tend to experience strong levels of distress) and openness (people high in openness have broad interests and seek experiences).

Big-Five personality traits can affect HRQoL of different disorders because they could influence patients’ willingness to take treatment options, predispose to neuropsychiatric symptoms and affect coping strategies [11]. Overall, conscientiousness was connected to higher compliance to treatments and indications of doctors, better physical and mental HRQoL, whereas neuroticism was linked to lower HRQoL [20–26].

Schoormans et al. [27] also showed that this adverse personality effect was limited to older men, suggesting that age should also be considered when exploring the relationship between personality traits and HRQoL in patients with other chronic diseases.

A similar type of inverse association between neuroticism and perceived health, showing how CKD patients with higher traits of neuroticism prove to be more preoccupied with their health symptoms, complaining about them more and consequently perceived poorer HRQoL [28].

Another relevant contribution by Poppe et al. [29] showed that CKD patients with neurotic personality tend to be less accepting of the disease and to endorse more denial. In support of this, the authors explain that high levels of neuroticism are usually associated with specific attitudes (inflexibility, withdrawal, passivity, wishful thinking, negative emotion focus, and less adaptive coping).

Beside this, the authors also hypothesised that the mental HRQoL of CKD patients can be directly explained by the relationship between neuroticism and the perception of health, as this association has been proven by many studies: neuroticism being associated with a worse perception of mental HRQoL [11, 24]. The authors explain that this negative association can probably be explained by the negative tendency of neurotic patients to be hyper-vigilant regarding the negative stimuli they encounter and excessively inattentive to the positive ones.

In addition, Ibrahim et al. [11] showed a positive association between extraversion and mental HRQoL, which is aligned with findings of other studies not strictly related to CKD patients [26]. The explanation given by the author is that extraverted people are more likely to be easily distracted away from their disabilities.

Moreover, conscientiousness, agreeableness and openness were not associated with HRQoL. However, since conscientiousness in other studies proved to be predictive of higher treatment adherence [24] and conscientiousness and agreeableness were associated with higher self-rated health [26].

### Illness denial in CKD

The concept of denial as recurrent defence mechanism in human experience was firstly introduced by Sigmund Freud (as cited in Rossi Ferrario et al. [30]) and subsequently refined by Anna Freud (as cited in Gagani et al. [31]) as an unintentional process which comes into play to reduce the anxiety caused by a specific threatening problem by preserving a person from something that he/she is not yet ready to face. For many years since this theorisation, denial has been considered mostly as uniquely pathological, but during the last times it has been recognised as playing a protective role in psychological functioning [32].

While generally considered as a unidimensional construct, other scholars suggested the complexity and fluctuation of denial and its negative and positive effects on HRQoL in chronic illness [33–35].

Nevertheless, a circular relationship was demonstrated between depression and negative self-care behaviours in CKD patients [30, 36], i.e., patients with depression are less likely to comply with medication, dialysis, and renal diet and more likely to have a sedentary lifestyle.

Recently, Rossi Ferrario et al. [30] proposed a new multidimensional model of denial and discussed its positive side as an effective strategy for facing the initial phases of an illness (i.e., invasive examinations or burdensome therapies), and the negative side when in its more severe, persistent forms, denial may lead to maladaptive behaviours and distress during the chronic course of the disease. The authors defined it as composed of two correlated components, namely denial of negative emotions (the emotional reactivity related to the individual’s emotional life and its regulation) and resistance to change (the behavioural efforts and life-style changes necessary to manage illness). The components represent a preliminary phase characterised by the removal of unpleasant material from consciousness. The authors also introduced a third independent component, the conscious avoidance, representing a later phase where awareness is present, but the individual voluntarily avoids facing the threatening situation.

In sum, following Gagani et al. [31], it is legitimate to assume that denial may be to some extent a functional strategy in the first phase of an illness, but it can prevent cure and control of chronic illness like CKD, particularly in the long run. Therefore, health professionals should verify whether CKD patients’ denial is adaptive or maladaptive so it can be addressed properly during treatment.

### Rationale and hypotheses of the present study

Low attention has been dedicated to the personality profiles and illness denial in CKD patients and their relation with the domains of HRQoL. Poorer HRQoL can predict a higher risk of hospitalization and mortality, and broadly a worse adjustment to the dialytic therapy. Thus, a clearer knowledge of the psychological variables associated with a worse HRQoL in the predialysis stage might suggest specific interventions in this population, with the aim of improving their adjustment to the new therapeutic pathway [11, 16].

The aim of the present study was to investigate the association between illness denial and Big-Five personality traits and HRQoL dimensions beyond the effect of gender, age, medical comorbidity (cardiovascular respiratory diseases and dysmetabolism) and psychological distress (i.e., trait anxiety) in a group of patients with CKD in the predialysis stage. We hypothesised that conscientiousness, agreeableness and openness are related to better physical, mental and social dimensions of HRQoL, and that neuroticism is related to a worse HRQoL in all its dimensions.

## Method

### Eligibility criteria and procedure

Participants were included if they met the criteria for a diagnosis of CKD, and they were in the pre-dialysis stage. Diagnosis of CKD was defined by indicators of kidney damage—imaging or proteinuria (i.e., albumin to creatinine ratio)—and decreased renal function (below thresholds of glomerular filtration rate estimated from serum creatinine concentration) for at least three months [8].

Participants were excluded if (a) medical diseases interfered with the completion of questionnaires (e.g., neurological diseases, psychiatric diseases, intellectual disabilities); (b) they were under 18 years of age; (c) they did not understand Italian at a sufficient level to complete the questionnaire. There were considered the parameters of creatinine, urinary creatinine, creatinine clearance and albumin in addition to age and gender, without the exclusion of any medical condition, because they were frequently associated with CKD [17]. When patients were recruited, at that time the outpatient clinic had patients with a GFR between 100 and 20. In addition, uricemia as an expression of associated urate dysmetabolism and levels of sodium were assessed to document possible electrolyte imbalances.

One hundred participants were recruited in an outpatient ward at the Santa Maria alle Scotte University Hospital of Siena, in Italy. Data was collected from March to September 2019. All the participants completed the questionnaires individually with the assistance of a psychologist, who provided information about the aims of the study. In accordance with the Ethical Principles of Psychologists and Code of Conduct, all the participants, who were included, provided written informed consent to participate in the study after having received a detailed description of the aims.

### Measures

#### Kidney Disease Quality of Life short form version 1.3 (KDQOL-SF) [37]

The KDQOL-SF 1 is a self-report questionnaire which consists of 11-item scales focusing on HRQoL issues specific to patients with CKD. These kidney disease-specific domains include List of Symptoms/Problems, Effects of Kidney Disease, Burden of Kidney Disease, Work Status, Cognitive Function, Quality of Social Interaction, Sexual Function, Sleep, Social Support. We linearly converted kidney disease-specific domain scores to a 0–100 scale in a similar manner to that used for the SF-36 domain scores. Higher scores suggest a higher perceived HR-QoL. A Kidney Disease Component Summary Score is generated as an average of these kidney disease-specific scales as previously reported [38]. In present study, the internal consistency of all the scales was good (range of Cronbach’s alpha = 0.82–0.86).

#### Italian version of Ten Item Personality Inventory Revised (I-TIPI-R) [39]

The I-TIPI-R is a 10-item scale, each consisting of a pair of descriptors that were scored from 1 (strongly disagrees) to 7 (strongly agree). Two items represented each dimension of the Big Five (Extraversion, Agreeableness, Conscientiousness, Neuroticism, Openness), one stated in a way that constitutes the positive pole of the dimension, and the other reported in a way that serves the negative pole. The measure showed acceptable psychometric properties in terms of test–retest reliability, factor structure, convergent validity with other personality questionnaires [39]. In  the current study, the internal consistency of all the scales was acceptable to good (range of Cronbach’s alpha = 0.76–0.82).

#### Illness Denial Questionnaire (IDQ) [30]

It consists of 24 dichotomous items (false = 0; true = 1) evaluating three dimensions: Denial of Negative Emotions (7 items; example item, “*I am worried about this disorder/disease*”); Resistance to Change (9 items; example item, “*The treatments (medications, exercises, or others) do not, in fact, change my life*”), and Conscious Avoidance (8 items; example item, “*I try to avoid thinking about this disorder/disease as much as I can*”). The first two dimensions express the core of denial, while the latter represents a more advanced stage of the illness elaboration process. Higher scores express higher denial levels. In present study, the internal consistency of all the scales was good (range of Cronbach’s alpha = 0.80–0.85).

#### State-Trait Anxiety Inventory-Y form (STAI-Y) [40]

The STAI-Y is a 20-item self-report tool. Each item is rated on a 4-point Likert scale with higher scores denoting higher levels of trait anxiety. Internal consistency of the STAI-Y was very good for the total community sample (Cronbach’s alpha = 0.89), and excellent for the total clinical sample (alpha = 0.90) In current study, the internal consistency of the scale was excellent (Cronbach’s alpha = 0.90).

### Statistical analysis

Pearson’s bivariate correlations were calculated between the KDQOL-SF scale scores and the STAI-Y Trait subscale, IDQ and I-TIPI-R scores. Values on the correlation coefficients were interpreted according to the following criteria [41]: 0 < *r* <|.30|= weak; |.30|< *r* <|.50|= moderate; |.50|< *r* <|.70|= strong; |.70|< *r* <|1|= very strong. Power calculations were run for this analysis: for a medium effect size, 80% power, and significance set at the level described above, the required sample size for bivariate correlations was at least 64 participants.

Subsequently, to test the specific contribution of anxious symptoms, illness denial processes and personality traits, generalised linear models were conducted entering age, gender (male vs. female), and the scores on the STAI-Y Trait subscale, IDQ and I-TIPI-R as predictors and the scores on each of the KDQOL-SF scales as dependent variables. The variables followed a normal distribution, therefore an identity link function was used. Power calculations were run for this analysis: for a medium effect size, 70% power, and significance set at the level described above, the required sample size for bivariate correlations was 100 participants. The statistical analysis was conducted using the SPSS software version 25.00 software.

## Results

### Descriptive characteristics

One hundred individuals with a diagnosis of CKD participated. Mean age was 75.87 years old (SD = 10.14, range = 42–94) and forty-one (41%) of the sample were females. The degree of CKD severity was assessed by collecting creatinine, creatinine clearance, and urea values. Descriptive socio-demographics, blood markers and scores on the questionnaires were reported in Table 1.

**Table 1:** Descriptive socio-demographics, CKD-related characteristics (blood markers) and scores on the questionnaires (*n* = 100)

	Minimum	Maximum	Mean	SD
*Socio-demographics characteristics*				
Mean age (years)	42	94	75.87	10.14
Gender	*n* (%)			
	Female (41%)			
	Male (59%)			
*Comorbidities*				
Dysmetabolism	*n* (%)			
	Yes (48%)			
	No (52%)			
Cardiovascular Disorders	Yes (71%)			
	No (29%)			
*Blood markers*				
Urea	38.000	203.000	90.940	37.358
Uric acid	2.700	11.300	5.246	1.685
Sodium	131.000	147.000	140.880	2.750
Creatinine Clearance	6.500	111.000	36.704	20.076
Creatinine	98.000	572.000	221.390	89.227
*Scores on the questionnaires*				
KDQOL-SF list of symptoms/problems	27.270	100.000	77.320	16.819
KDQOL-SF effects of kidney disease	46.875	100.000	85.071	12.636
KDQOL-SF burden of kidney disease	0	100.000	64.000	31.093
KDQOL-SF work status	0	100.000	49.500	21.899
KDQOL-SF cognitive function	20.000	100.000	78.133	21.400
KDQOL-SF quality of social interaction	40.000	100.000	74.600	18.488
KDQOL-SF sexual function	75.000	100.000	97.220	8.085
KDQOL-SF sleep	15.000	100.000	60.975	19.774
KDQOL-SF social support	0.000	100.000	71.166	28.214
KDQOL-SF kidney disease component summary score	10	100.000	60.300	18.719
STAI-Y Trait	20	75.000	40.900	10.490
IDQ denial of negative emotions	0	7.000	4.090	2.357
IDQ resistance to change	0	9.000	4.630	2.356
IDQ conscious avoidance	0	8.000	3.830	2.188
I-TIPI-R extraversion	2	14.000	7.910	2.871
I-TIPI-R agreeableness	2	14.000	10.300	2.634
I-TIPI-R conscientiousness	2	14.000	9.820	2.904
I-TIPI-R neuroticism	2	14.000	7.600	2.562
I-TIPI-R openness	2	14.000	6.960	2.420

*IDQ* Illness Denial Questionnaire, *I-TIPI-R* Italian Ten Item Personality Inventory Revised, *KDQOL-SF* Kidney Disease Quality of Life-Short Form, *STAI-Y* State-Trait Anxiety Inventory-Y form

### Associations between quality of life, illness denial and personality traits

The results of the correlational analyses are presented in Table 2. Scores on the IDQ Denial of Negative Emotions correlated moderately and positively with scores on the KDQOL-SF Effects of Kidney Disease, KDQOL-SF Burden of Kidney Disease, and KDQOL-SF Cognitive Function, and weakly with scores on the KDQOL-SF List of Symptoms/Problems and KDQOL-SF Sleep.

**Table 2:** Pearson’s correlation coefficients (*n* = 100)

	STAI-Y Trait	IDQ Denial of Negative Emotions	IDQ Resistance to Change	IDQ Conscious Avoidance	I-TIPI-R Extraversion	I-TIPI-R Agreeableness	I-TIPI-R Conscientiousness	I-TIPI-R Neuroticism	I-TIPI-R Openness
KDQOL-SF list of symptoms/problems	− .415**	.241^*^	.229^*^	− .162	.069	.048	.098	− .102	.262^**^
KDQOL-SF effects of kidney disease	− .289**	.406**	.332**	− .048	.098	.084	.144	− .192	.135
KDQOL-SF burden of kidney disease	− .179	.412**	.337**	− .035	− .001	.090	− .014	− .035	.153
KDQOL-SF work status	.013	.060	.084	− .107	.192	− .050	.197^*^	.104	.047
KDQOL-SF cognitive function	− .423**	.430**	.336**	− .200^*^	.197^*^	.135	.145	− .074	.079
KDQOL-SF quality of social interaction	− .282**	.169	.187	− .080	.109	.247^*^	.021	− .066	− .011
KDQOL-SF sexual function	− .295**	.194	.423	− .210	− .036	.142	− .045	− .032	.122
KDQOL-SF sleep	− .381**	.240^*^	.137	.055	.080	.086	− .052	− .242^*^	.084
KDQOL-SF social support	− .253^*^	.196	.046	− .263**	.186	.059	− .093	− .056	− .106
KDQOL-SF kidney disease component summary score	− .331**	.189	.190	− .169	.145	.152	.016	− .044	.054

*IDQ* Illness Denial Questionnaire, *I-TIPI-R* Italian Ten Item Personality Inventory Revised, *KDQOL-SF* Kidney Disease Quality of Life-Short Form, *STAI-Y* State-Trait Anxiety Inventory-Y form

**p < .01, *p < .05

Positive and moderate associations emerged between scores on the IDQ Resistance to Change and scores on the KDQOL-SF Effects of Kidney Disease, KDQOL-SF Burden of Kidney Disease and KDQOL-SF Cognitive Function. Scores on the IDQ Conscious Avoidance were negatively and weakly related to scores on the KDQOL-SF Cognitive Function and KDQOL-SF Social Support. Scores on the KDQOL-SF Cognitive Function were associated positively and weakly with scores on I-TIPI-R Extraversion; scores on the KDQOL-SF Quality of Social Interaction correlated positively and weakly with scores on I-TIPI-R Agreeableness. Scores on the KDQOL-SF Work Status correlated positively and weakly with scores on I-TIPI-R Conscientiousness. Negative and weak correlations were found between scores on the KDQOL-SF Sleep and scores on I-TIPI-R Neuroticism. Scores on KDQOL-SF List of Symptoms/Problems correlated positively and weakly with scores on I-TIPI-R Openness.

### Illness denial and personality traits as predictors of HRQoL

**Fig. 1:**
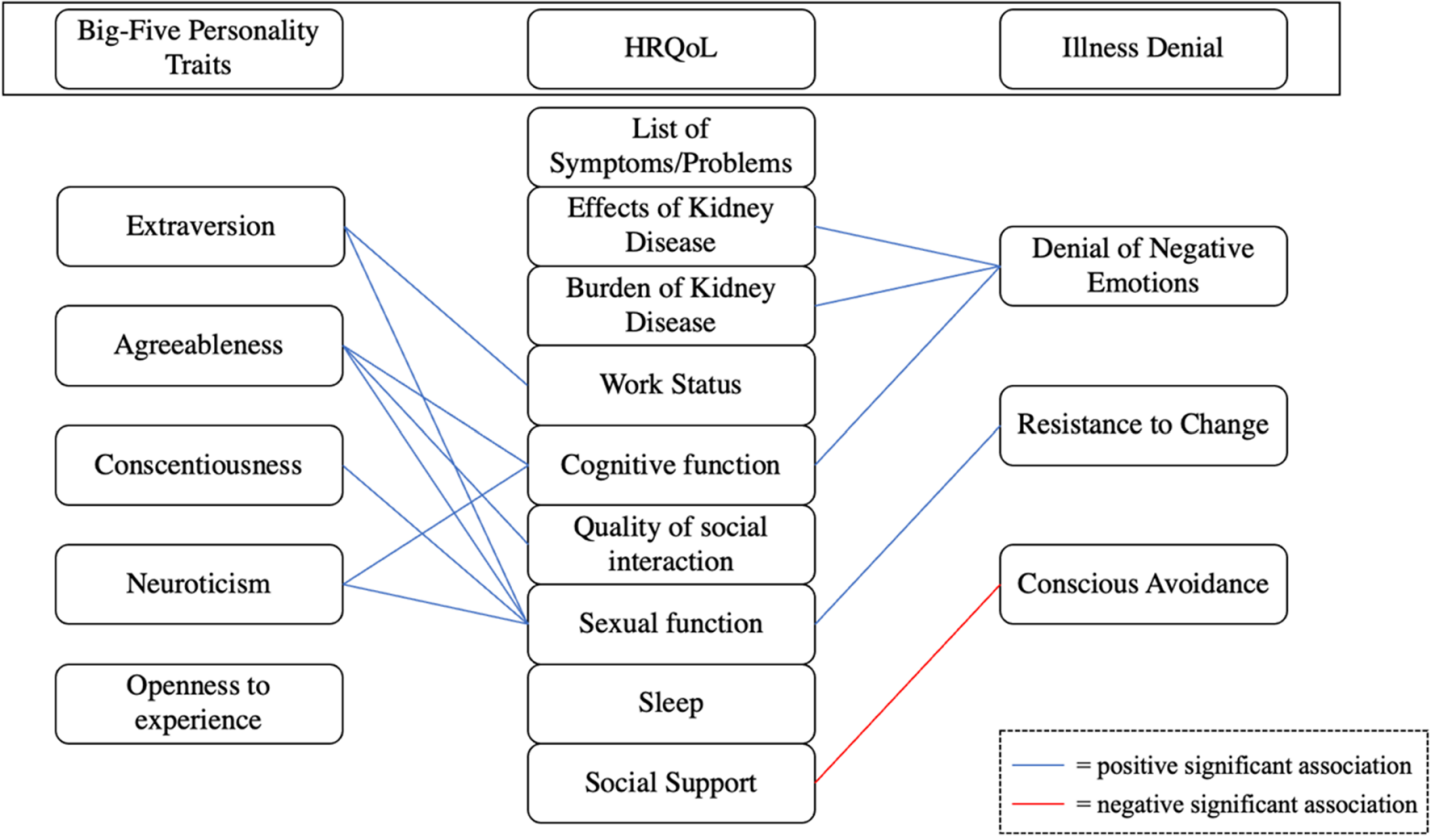
Significant associations between Personality Traits, Illness Denial and HRQoL through generalised linear models

The results of the generalised linear models are presented in Table 3 and illustrated in Fig. 1. Scores on the STAI-Y Trait and I-TIPI-R Openness negatively (*B* = − 0.653; *p* = 0.000) and positively (*B* = 1.597; *p* = 0.009) predicted scores on the KDQOL-SF List of Symptoms/Problems respectively. Scores on the IDQ Denial of Negative Emotions positively predicted scores on the KDQOL-SF Effects of Kidney Disease (*B* = 1.536; *p* = 0.031). Age and scores on the IDQ Denial of Negative Emotions negatively (*B* = − 0.928; *p* = 0.001) and positively (*B* = 6.017; *p* = 0.000) predicted scores KDQOL-SF Burden of Kidney Disease respectively. Age and scores on the I-TIPI-R Extraversion negatively (*B* = − 1.113; *p* = 0.000) and positively (*B* = 1.591; *p* = 0.016) predicted scores on KDQOL-SF Work Status. The presence of dysmetabolism was associated with higher scores on the KDQOL-SF Work Status. Scores on the KDQOL-SF Cognitive Function were positively predicted by scores on the IDQ Denial of Negative Emotions (*B* = 3.766; *p* = 0.000), I-TIPI-R Agreeableness (*B* = 1.609; *p* = 0.009), I-TIPI-R Neuroticism (*B* = 1.884; *p* = 0.007) and negatively predicted by age and STAI-Y Trait (*B* = -0.708; *p* = 0.000). Female gender (*B* = 9.520; *p* = 0.008) and scores on the I-TIPI-R Agreeableness (*B* = 1.425; *p* = 0.036) positively predicted scores on the KDQOL-SF Quality of Social Interaction while scores on the STAI-Y Trait were negative predictors of this KDQOL-SF scale (*B* = − 0.523; *p* = 0.006). Scores on the KDQOL-SF Sexual Function were positively predicted by female gender (*B* = 14.468; *p* = 0.000) and scores on the IDQ Resistance to Change (*B* = 4.865; *p* = 0.000), I-TIPI-R Extraversion (*B* = 1.729; *p* = 0.000), I-TIPI-R Agreeableness (*B* = 1.286; *p* = 0.000), I-TIPI-R Conscientiousness (*B* = 0.950; *p* = 0.000), and I-TIPI-R Neuroticism (*B* = 0.759; *p* = 0.000), and negatively by age (*B* = − 0.553; *p* = 0.000). Scores on the KDQOL-SF Social Support were negatively predicted by scores on the IDQ Conscious Avoidance (*B* = -2.980; *p* = 0.011). Scores on the KDQOL-SF Kidney Disease Component Summary Score were predicted negatively by scores on the STAI-Y Trait (*B* = − 0.649; *p* = 0.001) and cardiovascular respiratory diseases (*B* = − 9.801; *p* = 0.013).

**Table 3:** Generalised linear models of socio-demographic and clinical predictors of KDQOL scores (n = 100)

		95% CI			
	*B*	Lower	Upper	Wald’s *χ*^*2*^_*(1)*_	*p*-value
*Outcome: KDQOL-SF list of symptoms/problems*					
Intercept	101.897	66.975	136.820	32.705	.000
Females	− 5.184	− 11.263	.895	2.793	.095
Males	0^a^				
Age (years)	− .271	− .569	.027	3.172	.075
Cardiovascular respiratory diseases	− 2.086	− 8.756	4.584	.376	.540
Dysmetabolism	.592	− 5.270	6.454	.039	.843
STAI-Y Trait	− .653	− .979	− .327	15.394	.000
IDQ denial of negative emotions	.574	− 1.186	2.334	.408	.523
IDQ resistance to change	.332	− 1.344	2.008	.151	.698
IDQ conscious avoidance	− .964	− 2.260	.333	2.123	.145
I-TIPI-R extraversion	− .247	− 1.279	.786	.219	.640
I-TIPI-R agreeableness	.671	− .484	1.826	1.296	.255
I-TIPI-R conscientiousness	.019	− .996	1.035	.001	.970
I-TIPI-R neuroticism	1.242	− .068	2.552	3.456	.063
I-TIPI-R openness	1.597	.405	2.789	6.892	.009
*Outcome: KDQOL-SF effects of kidney disease*					
Intercept	68.966	41.284	96.648	23.844	.000
Females	2.528	− 2.291	7.346	1.057	.304
Males	0^a^				
Age (years)	.056	− .180	.293	.220	.639
Cardiovascular respiratory diseases	− 2.511	− 7.798	2.776	.866	.352
Dysmetabolism	1.704	− 2.942	6.351	.517	.472
STAI-Y Trait	− .156	− .415	.102	1.405	.236
IDQ denial of negative emotions	1.536	.141	2.931	4.657	.031
IDQ resistance to change	.380	− .949	1.709	.314	.575
IDQ Conscious Avoidance	.097	− .931	1.124	.034	.854
I-TIPI-R Extraversion	.169	− .650	.988	.164	.686
I-TIPI-R agreeableness	.028	− .887	.944	.004	.952
I-TIPI-R conscientiousness	.481	− .323	1.286	1.375	.241
I-TIPI-R neuroticism	− .119	− 1.157	.920	.050	.823
I-TIPI-R openness	.571	− .374	1.516	1.402	.236
*Outcome: KDQOL-SF burden of kidney disease*					
Intercept	76.422	11.214	141.629	5.276	.022
Females	7.293	− 4.057	18.644	1.586	.208
Males	0^a^				
Age (years)	− .928	− 1.484	− .371	10.674	.001
Cardiovascular respiratory diseases	4.128	− 8.326	16.582	.422	.516
Dysmetabolism	5.766	− 5.180	16.711	1.066	.302
STAI-Y Trait	− .188	− .797	.422	.364	.546
IDQ denial of negative emotions	6.017	2.731	9.304	12.879	.000
IDQ resistance to change	.415	− 2.715	3.544	.067	.795
IDQ Conscious Avoidance	.796	− 1.624	3.217	.416	.519
I-TIPI-R extraversion	− .122	− 2.050	1.807	.015	.902
I-TIPI-R agreeableness	1.816	− .341	3.972	2.723	.099
I-TIPI-R conscientiousness	− 1.256	− 3.151	.639	1.687	.194
I-TIPI-R neuroticism	1.768	− .678	4.214	2.007	.157
I-TIPI-R openness	1.077	− 1.150	3.303	.899	.343
*Outcome: KDQOL-SF work status*					
Intercept	80.338	36.396	124.280	12.841	.000
Females	6.622	− 1.026	14.271	2.880	.090
Males	0^a^				
Age (years)	− 1.113	− 1.488	− .738	33.839	.000
Cardiovascular respiratory diseases	5.411	− 2.982	13.803	1.597	.206
Dysmetabolism	8.200	.824	15.576	4.748	.029
STAI-Y Trait	.211	− .200	.622	1.015	.314
IDQ denial of negative emotions	1.920	− .295	4.134	2.886	.089
IDQ resistance to change	− .161	− 2.270	1.948	.022	.881
IDQ conscious avoidance	− .754	− 2.385	.877	.821	.365
I-TIPI-R extraversion	1.591	.291	2.891	5.757	.016
I-TIPI-R agreeableness	.501	− .952	1.954	.456	.499
I-TIPI-R conscientiousness	.678	− .599	1.956	1.084	.298
I-TIPI-R Neuroticism	.956	− .692	2.604	1.293	.256
I-TIPI-R Openness	− .412	− 1.912	1.089	.289	.591
*Outcome: KDQOL-SF cognitive function*					
Intercept	122.271	85.752	158.791	43.062	.000
Females	5.403	− .954	11.759	2.775	.096
Males	0^a^				
Age (years)	− .851	− 1.163	− .539	28.621	.000
Cardiovascular respiratory diseases	− 5.725	− 12.700	1.250	2.588	.108
Dysmetabolism	5.101	− 1.029	11.231	2.660	.103
STAI-Y Trait	− .708	− 1.049	− .367	16.539	.000
IDQ denial of negative emotions	3.766	1.926	5.607	16.088	.000
IDQ resistance to change	− .177	− 1.930	1.575	.039	.843
IDQ conscious avoidance	− .926	− 2.282	.430	1.791	.181
I-TIPI-R Extraversion	.840	− .240	1.920	2.324	.127
I-TIPI-R Agreeableness	1.609	.401	2.817	6.820	.009
I-TIPI-R conscientiousness	− .105	− 1.166	.957	.038	.846
I-TIPI-R neuroticism	1.884	.514	3.254	7.265	.007
I-TIPI-R Openness	.044	− 1.202	1.291	.005	.944
*Outcome: KDQOL-SF quality of social interaction*					
Intercept	77.835	37.610	118.059	14.383	.000
Females	9.520	2.519	16.522	7.102	.008
Males	0^a^				
Age (years)	− .076	− .419	.267	.189	.664
Cardiovascular respiratory diseases	− 3.431	− 11.114	4.252	.766	.381
Dysmetabolism	5.909	− .843	12.661	2.942	.086
STAI-Y Trait	− .523	− .899	− .148	7.452	.006
IDQ denial of negative emotions	.201	− 1.826	2.228	.038	.846
IDQ resistance to change	.578	− 1.352	2.509	.345	.557
IDQ conscious avoidance	− .307	− 1.800	1.187	.162	.687
I-TIPI-R extraversion	.532	− .657	1.722	.769	.381
I-TIPI-R agreeableness	1.425	.094	2.755	4.406	.036
I-TIPI-R conscientiousness	− .664	− 1.833	.505	1.240	.266
I-TIPI-R neuroticism	.568	− .941	2.077	.544	.461
I-TIPI-R Openness	− .061	− 1.434	1.313	.007	.931
*Outcome: KDQOL-SF sexual function*					
Intercept	45.279	30.774	59.785	37.430	.000
Females	14.468	10.541	18.396	52.131	.000
Males	0^a^				
Age (years)	− .533	− .624	− .443	133.794	.000
Cardiovascular respiratory diseases	.862	− 1.987	3.711	.352	.553
Dysmetabolism	15.319	13.329	17.308	227.772	.000
STAI-Y Trait	.060	− .099	.219	.541	.462
IDQ Denial of Negative Emotions	− .577	− 1.267	.113	2.689	.101
IDQ Resistance to change	4.865	4.037	5.694	132.515	.000
IDQ conscious avoidance	.253	− .213	.719	1.134	.287
I-TIPI-R extraversion	1.729	.959	2.500	19.337	.000
I-TIPI-R agreeableness	1.286	.762	1.811	23.105	.000
I-TIPI-R conscientiousness	.950	.546	1.353	21.279	.000
I-TIPI-R Neuroticism	.759	.425	1.092	19.873	.000
I-TIPI-R Openness	− .049	− 1.097	.999	.008	.927
*Outcome: KDQOL-SF sleep*					
Intercept	79.792	37.214	122.370	13.491	.000
Females	7.072	− .340	14.483	3.497	.061
Males	0^a^				
Age (years)	.065	− .299	.428	.122	.727
Cardiovascular respiratory diseases	6.700	− 1.432	14.832	2.607	.106
Dysmetabolism	− 5.136	− 12.283	2.011	1.984	.159
STAI-Y Trait	− .592	− .990	− .194	8.508	.004
IDQ denial of negative emotions	1.858	− .288	4.004	2.880	.090
IDQ resistance to change	− 1.249	− 3.292	.795	1.434	.231
IDQ conscious avoidance	1.160	− .420	2.741	2.070	.150
I-TIPI-R extraversion	.519	− .740	1.779	.653	.419
I-TIPI-R Agreeableness	.149	− 1.259	1.557	.043	.835
I-TIPI-R conscientiousness	− .975	− 2.213	.262	2.385	.122
I-TIPI-R neuroticism	− .865	− 2.462	.732	1.126	.289
I-TIPI-R openness	.064	− 1.389	1.518	.008	.931
*Outcome: KDQOL-SF social support*					
Intercept	81.998	20.471	143.525	6.823	.009
Females	− 5.310	− 16.020	5.400	.944	.331
Males	0^a^				
Age (years)	.233	− .292	.758	.755	.385
Cardiovascular respiratory diseases	− 5.952	− 17.704	5.799	.986	.321
Dysmetabolism	4.977	− 5.350	15.305	.892	.345
STAI-Y Trait	− .748	− 1.323	− .173	6.507	.011
IDQ denial of negative emotions	1.981	− 1.120	5.081	1.567	.211
IDQ resistance to change	− 1.697	− 4.650	1.256	1.268	.260
IDQ conscious avoidance	− 2.980	− 5.264	− .695	6.537	.011
I-TIPI-R extraversion	1.581	− .238	3.401	2.901	.089
I-TIPI-R agreeableness	1.054	− .981	3.089	1.031	.310
I-TIPI-R conscientiousness	− 1.014	− 2.802	.774	1.235	.266
I-TIPI-R neuroticism	1.445	− .863	3.753	1.506	.220
I-TIPI-R openness	− 1.150	− 3.251	.950	1.152	.283
*Outcome: KDQOL-SF Kidney disease component summary score*					
Intercept	76.649	35.994	117.303	13.655	.000
Females	− 3.026	− 10.103	4.050	.703	.402
Males	0^a^				
Age (years)	− .098	− .445	.249	.308	.579
Cardiovascular respiratory diseases	− 9.801	− 17.566	− 2.036	6.121	.013
Dysmetabolism	− .963	− 7.787	5.861	.076	.782
STAI-Y Trait	− .649	− 1.029	− .269	11.210	.001
IDQ denial of negative emotions	− .049	− 2.097	2.000	.002	.963
IDQ resistance to change	.969	− .982	2.920	.948	.330
IDQ conscious avoidance	− 1.231	− 2.740	.278	2.555	.110
I-TIPI-R extraversion	.356	− .847	1.558	.336	.562
I-TIPI-R agreeableness	1.329	− .016	2.673	3.751	.053
I-TIPI-R conscientiousness	− .428	− 1.610	.754	.503	.478
I-TIPI-R neuroticism	1.479	− .046	3.004	3.611	.057
I-TIPI-R openness	.484	− .904	1.872	.468	.494

*CI* Confidence Interval; *IDQ* Illness Denial Questionnaire; *I-TIPI-R* Italian Ten Item Personality Inventory Revised; *KDQOL-SF* Kidney Disease Quality of Life-Short Form; *STAI-Y* State-Trait Anxiety Inventory-Y form

^a^Parameter set at 0 because redundant in the model

## Discussion

The present work is the first investigation which simultaneously assessed Big-Five personality traits and illness denial in the different domains of HRQoL of CKD patients in the predialysis stage. The results showed that different illness denial dimensions and Big-Five personality traits have a specific role in specific HRQoL dimensions of CKD.

### Key findings

Illness denial was associated with increased HRQoL in symptoms, effect, burden of kidney disease and cognitive functions domains, and it was a predictor of higher HRQoL in the last three domains mentioned above. Extraversion was related to better work status and sexual function; agreeableness was linked to elevated cognitive function, quality of social interaction and sexual function; conscientiousness was related to better sexual function; neuroticism was linked to improved cognitive and sexual functions; in the end, openness to experience was related to fewer symptoms and problems.

### The role of illness denial in HRQoL of CKD patients

Denial of negative emotions and resistance to change were found to be associated with the same dimensions of HRQoL. Individuals with high denial of negative emotions and resistance to change tended to report higher quality of life related to symptom/problems, the effect of kidney disease, the burden of kidney disease and cognitive function. Such findings seem to be the evidence that these dimensions of denial might represent an actual expression of denial, whereas conscious avoidance seems to constitute a different step in the process of cognitive-affective processing of the illness [30]. In addition, higher denial of negative emotions was related to a more elevated HRQoL linked to the disease effect, burden and cognitive function.

Therefore, individuals who reported elevated levels of denial tended to claim that they were not bothered by the effects of the kidney disease on daily life (e.g., restriction on fluid, dietary intake, impact on work etc.), did not perceive high levels of frustration and interference of kidney disease in their life, and did not report any concentration problems or mental confusion. It may be the one’s tendency to deny negative emotions, which arises from the effect and burden of CKD, making people report that they experience a better HRQoL in the above-mentioned areas. Being aware of one’s own distancing from the illness may represent not only the point in the denial process at which acceptance of the illness’s existence begins [30], but also it may be an effective method for facing the phases of the illness and improving the perceived HRQoL. It can be also speculated that the tendency to illness denial, making subjects feel not to be sick, protected them from a worsening of their HRQoL.

The finding that people with higher conscious avoidance had lower levels of social support suggests that individuals with a greater tendency to avoid and take distance from the real condition were less satisfied with their social support. People who asserted, for example, that “less I know, the better I feel” or “I try not to speak about this disorder/disease” might feel like they did not need the support of their friends or family; social support could be actually a way to make the illness more real and close. Nevertheless, individuals who do not perceive adequate social support could tend to voluntarily avoid facing the threatening situation, having already awareness of the illness. That could imply that social support would be a resource for the individual who is trying to cope with kidney disease.

### The role of Big-Five personality traits in HRQoL of CKD patients

Assessing the correlation between personality traits and HRQoL means comparing a construct that is relatively stable (the first) with a construct that can change over time; which means that the relationship between those construct may change.

Extraversion was associated with positive cognitive function, in line with the evidence which highlighted a relation between extraversion and better mental health in patients with CKD [11] and with findings, in kidney transplant recipients, indicating that extroverted people are more likely to be distracted away from disabilities by focusing on external stimulation and by engaging in daily activities [28]. Quality of social interaction was related to agreeableness, suggesting that amiable patients with CKD have better social support, in line with previous research [11, 29]. People with higher conscientiousness tended to report to be more capable of working. This could be explained as conscientiousness, being related to greater adherence to treatment [29], could be indirectly linked with a better HRQoL and thus, indirectly, a better work performance and status. The trait of openness to experience was related to and was a predictor of a greater HRQoL concerning symptoms/problems, in contrast with the literature on general population where openness seems to be unrelated to self-rated health [26]. This could mean that individuals who are more “open to experience” might be less in touch with their inner body signals and thus be less negatively focused on their symptoms [42]. Traits of agreeableness and neuroticism both predicted a better cognitive function. Consistently with a previous study [26], agreeableness predicted a better self-rated health, but only one study found a relation between agreeableness and quality of life in patients with chronic renal failure [43]. In contrast with several studies in general population [30], in kidney transplant recipients [43] and in CKD patients [29, 44], in our investigation neuroticism was found to be a predictor of a better quality of life in the cognitive function domain. Individuals with higher neuroticism were likely to be more concerned about the illness and hyper-vigilant towards negative stimuli that they encountered. Patients with neurotic traits may report better cognitive functioning to be believed in their complaints about their symptoms, in line with Ferentzi et al. [42]. Patients with higher denial of the disease could claim to have a good cognitive function. We could also speculate that individuals with high neuroticism would tend to seek a lot of information. This could result in higher levels of health literacy, which together with low levels of cognitive impairment were associated with increased quality of life [17, 45]. Lastly, extraversion, agreeableness, conscientiousness and neuroticism were predictors of a greater sexual function.

### Role of socio-demographic and clinical variables

This study reports important effects of some socio-demographic predictors on the HRQoL of individuals with CKD. First, older age was found to be a predictor of a worse HRQoL related to the burden of kidney disease, work status, cognitive and sexual functions. The interpretation of this finding, which is consistent with previous studies [11, 46], should take into account that older age implies a longer duration of illness and contextually there may be an amount of other factors that could influence the quality of life of the patients. Feminine gender was a predictive factor of a better HRQoL in the domains of sexual function and quality of social interaction. Therefore, being a woman seems to be associated with a lower impact of kidney disease on individual’s sexual functioning and social isolation. The comorbidity with cardiovascular respiratory disease, which is frequent in patients with CKD [17], was a predictor of worse general HRQoL. In fact, comorbidity reduces chances of survival and increases hospitalisation [17]. Dysmetabolism was a predictor of a better HRQoL, mostly impacting the work status and sexual function. Lastly, trait anxiety was negatively associated with and was a negative predictor of nearly all the dimensions of the HRQoL, indicating that this stable trait of personality, characterised by high reactivity to stimulation and high arousal [47], predicted lower perception of HRQoL.

### Limitations and future directions

The cross-sectional study design did not allow us to reliably ascertain the role of illness denial and Big Five traits as risk factors for a worse HRQoL. Furthermore, in our case, multiple testing could imply a large probability that some of the true null hypotheses will be rejected, thus resulting in type I error. In addition, a multicentre design could increase the generalisability of the findings to different healthcare settings. Another limitation concerned the use of self-report questionnaires, which should be combined with future interviews made by clinicians.

Although the association between illness denial and HRQoL we has been shown in the present work, the results could be interpreted that denial attitude affected only responses to the questionnaire, rather than affecting HRQoL itself. Nevertheless, since HRQoL is a much broader construct, it should be investigated by further, more objective measures. Thereby, the association would also be strengthened. Furthermore, since personality traits are a relatively stable construct over time [18, 48], whereas the HRQoL may change, forthcoming studies should use a longitudinal design to explore this dynamic relationship over time.

Duration of illness was not evaluated, and additional markers were not considered due to lack of laboratory data at the time of questionnaire administration. In addition, future research should take into account further covariates related to blood markers such as proteinuria, anaemia, hemoglobin levels, siderosis, and potassium levels that can be associated with illness severity. However, we did not have enough statistical power due to the relatively small sample size.

Furthermore, it could be helpful to consider the relationships between the above evidence and the state of depression in CKD patients. In fact, from past studies, depression was found to be common amongst the patients with chronic physical health problems [49, 50], and in those affected by CKD depression levels seem to be even higher than that reported levels for the patients with other chronic diseases [51]. Another variable that might be involved as a moderator of the relations between illness denial/personality traits and HRQoL could be the stage of the disease, i.e., being or not on haemodialytic therapy. Additional variables may also include further social and demographic features that can negatively impact on health literacy and access to healthcare services such as immigrant status which has been found to be a predictor of several chronic diseases [52–54].

It would be interesting, in the future, to investigate the possible correlation between the personality trait openness and a greater perception of one's disease or, vice versa, a judgemental attitude towards one’s disease. Further studies based on a larger sample size would be warranted to expand the knowledge on this subject, in the belief that better acquisitions can lead to specific and personalised interventions both from a physical and mental point of view, as found for other chronic diseases [55, 56]. Finally, future research should explore more in depth the inter-relationships between the predictors by testing interaction effects through moderation analysis in larger samples. In addition, further studies based on larger samples should identify subgroups of patients on specific clinical and psychological features by latent profile analysis.

## Conclusions

To our knowledge, this is the first study which simultaneously assessed Big-Five personality traits and illness denial in different domains of HRQoL of CKD patients. Illness denial was associated with increased HRQoL related to symptoms/problems, effect and burden of kidney disease and cognitive functions domains, and it was a predictor of higher HRQoL in the last three domains mentioned above. Extraversion was related to better work status and sexual function; agreeableness was linked to elevated cognitive function, quality of social interaction and sexual function; conscientiousness was related to better sexual function; neuroticism was linked to improved cognitive and sexual functions; in the end, openness to experience was related to fewer symptoms and problems.

## Data Availability

The datasets used and/or analysed during the current study are available from the corresponding author on reasonable request.

## Abbreviations

CKD: Kidney Chronic Disease
HRQoL: Health-Related Quality of Life
KDQOL-SF: Kidney Disease Quality of Life Short form
I-TIPI-R: Ten Item Personality Inventory Revised
IDQ: Illness Denial Questionnaire
STAI-Y: State-Trait Anxiety Inventory Form-Y
